# On the Complexity of Brain Disorders: A Symptom-Based Approach

**DOI:** 10.3389/fncom.2016.00016

**Published:** 2016-02-23

**Authors:** Ahmed A. Moustafa, Joseph Phillips, Szabolcs Kéri, Blazej Misiak, Dorota Frydecka

**Affiliations:** ^1^School of Social Sciences and Psychology, Western Sydney UniversitySydney, NSW, Australia; ^2^Marcs Institute for Brain and Behavior, Western Sydney UniversitySydney, NSW, Australia; ^3^Nyírö Gyula Hospital, National Institute of Psychiatry and AddictionsBudapest, Hungary; ^4^Department and Clinic of Psychiatry, Wroclaw Medical UniversityWroclaw, Poland; ^5^Department of Genetics, Wroclaw Medical UniversityWroclaw, Poland

**Keywords:** brain disorders, functional connectivity, neurotransmitters, regional brain volume, major depressive disorder, Parkinson’s disease, schizophrenia, posttraumatic stress disorder

## Abstract

Mounting evidence shows that brain disorders involve multiple and different neural dysfunctions, including regional brain damage, change to cell structure, chemical imbalance, and/or connectivity loss among different brain regions. Understanding the complexity of brain disorders can help us map these neural dysfunctions to different symptom clusters as well as understand subcategories of different brain disorders. Here, we discuss data on the mapping of symptom clusters to different neural dysfunctions using examples from brain disorders such as major depressive disorder (MDD), Parkinson’s disease (PD), schizophrenia, posttraumatic stress disorder (PTSD) and Alzheimer’s disease (AD). In addition, we discuss data on the similarities of symptoms in different disorders. Importantly, computational modeling work may be able to shed light on plausible links between various symptoms and neural damage in brain disorders.

## Introduction

Brain disorders are associated with several symptom clusters as well as many neural dysfunctions. For example, although Parkinson’s disease (PD) is associated with reduced dopamine levels in the brain (Kish et al., 1988), studies found other neural dysfunctions including abnormal subthalamic nucleus oscillations as well as changes in cortical and cerebellar structures (Levy et al., 2000; Wu and Hallett, 2013). Further, PD is associated with different classes of motor symptoms, including akinesia, bradykinesia, tremor, and medication-induced dyskinesia. These symptoms are caused by a somewhat different neural abnormalities (which we will discuss this below). The same applies to Alzheimer’s disease (AD) as it involves many symptom clusters (including memory loss, apraxia, language impairment, and executive dysfunction) and multiple neural abnormalities including damage in the hippocampus and neocortex (Wilcock and Esiri, 1982). Similarly, psychiatric disorders are characterized by various psychopathological symptoms with complex underlying neural substrates. Major depressive disorder (MDD), for example, is associated with mood, physical, and cognitive changes, among other behavioral symptoms (Nutt et al., 2007). At the neural level, MDD symptoms are associated withreduced levels of dopamine and serotonin as well as decreased volumes of the hippocampus and prefrontal cortex (Drevets et al., 2008). Similarly (and rather unsurprisingly), posttraumatic stress disorder (PTSD) and schizophrenia are associated with various symptom clusters (i.e., positive vs. negative symptoms in schizophrenia, and avoidance vs. re-experiencing in PTSD); both disorders are associated with widespread neural damage. Unlike MDD and PD, there have been a larger number of studies that attempt to map schizophrenia symptoms to dissociable neural substrates (Wolkin et al., 1992; Buchanan et al., 1993; Okubo et al., 1997; Abi-Dargham, 2003; Rueter et al., 2004; Cascella et al., 2008; Farkas et al., 2008; Kéri, 2008; Polgár et al., 2010; Arnedo et al., 2015).

Below, we discuss various symptom clusters, what we know about their neural substrates, as well as potential computational modeling work to help understand behavioral-neural relationships in the following brain disorders: MDD, PD, AD, PTSD and schizophrenia.

## Computational Modeling

For many years now, the importance of dysfunctional neural circuits and/or brain regions is being underlined when considering the pathophysiological mechanisms underpinning psychopathological symptoms of neuropsychiatric disorders. However, inter- and intra-regional neural connections and disconnections are still poorly understood at the present time. In order to shed more light on the neurobiological substrates of neuropsychiatric symptomatology, computational models rooted in translational cognitive neuroscience are being created in order to explain variety of behavioral, neurophysiological and neuroanatomical data (Eliasmith et al., 2012; Rasmussen and Eliasmith, 2013). Computational models usually consist of reciprocally connected loops between different circuits with top-down and bottom-up signaling at the cortical and subcortical levels (McClelland et al., 1995; O’Reilly and Norman, 2002; Bobier et al., 2014). In the current literature, the most commonly applied models are reinforcement learning models – allowing to analyze key aspects of choice, and Bayesian models – formalizing main aspects of the inference prior beliefs with current sensory data, each weighted according to their uncertainty (Pauli et al., 2012). Recent developments in this area demonstrate that such models hold potential to expand our understanding of neurologic and psychiatric disorders (Frank et al., 2012; Adams et al., 2016).

## Major Depressive Disorder

MDD is a psychiatric disorder characterized by reduced mood, anhedonia, psychomotor retardation, and learned helplessness, among others (Kennedy, 2008). It is known that MDD involves changes to different neurotransmitters, while the most salient change is to serotonin. There are also changes to dopaminergic and noradrenergic transmission (Nutt, 2008). MDD is also associated with changes to brain volumes and connectivity networks among different brain regions. For example, studies have reported reduced gray matter volume in the anterior cingulate (van Tol et al., 2010) and hippocampus (Videbech and Ravnkilde, 2004) in patients with MDD. Other studies reported increased connectivity among the anterior cingulate and medial temporal lobe (de Kwaasteniet et al., 2013), while others reported a decrease in the functional connectivity in the insula and amygdala (Veer et al., 2010). These findings highlight the fact that MDD is associated with multiple neural dysfunctions. These previously mentioned studies did not, however, map these neural abnormalities to specific symptoms in depression. However, there are some studies that have attempted to understand the neural correlates for each symptom in depression (Vrieze et al., 2014). For example, Argyropoulos and Nutt (2013) found that anhedonia (the inability to experience pleasure) is related to dopamine reduction, while reduced mood is related to decreased serotonin levels. Some studies have investigated the neural substrates of psychomotor retardation (lack of energy and reduced movement) in depression, and suggested that reduced dopamine is also implicated (Liberg and Rahm, 2015). It is possible that reduced dopamine levels in the ventral striatum is related to anhedonia whereas reduced dopamine levels in the dorsal striatum is related to psychomotor retardation (Stein, 2008). In addition, it was suggested that reduced mood and sadness in MDD is associated with dysfunction in the prefrontal cortex, particularly, the orbitofrontal cortex (Drevets, 1999; Mayberg et al., 1999; Davidson et al., 2002; Drevets et al., 2002; Lévesque et al., 2003). Although the studies mentioned above have mapped certain symptoms of MDD to separable neural dysfunctions, the exact mechanism of these observations remains unclear. Computational modeling work should attempt to explain the behavioral-neural relationships, such as how a reduction of dopamine in the striatum leads to psychomotor retardation.

The concept that depression is accompanied by a dysfunctional reward system has been shown empirically numerous times over past years (for review, see Chen et al., 2015). Using prediction error learning algorithm to model behavioral performance in MDD, Steele et al. (2007) have shown that speeding of reaction times after wins and slowing after loses were not as pronounced as in the healthy control subjects. Moreover, this feedback-related speeding/slowing effect was associated with anhedonia. In the meta-analytic study performed by Huys et al. (2013) showed that anhedonia in depressive states is mediated by a change in reward sensitivity, which is different from either stress or dopamine manipulations, such as receiving dopamine D2 agonists. Additionally, he has shown that anhedonia affect appetitive learning more by reducing the primary sensitivity to rewards than by affecting the learning rates.

## Parkinson’S Disease

PD is characterized by tremor, akinesia, rigidity, bradykinesia, and gait dysfunction among other symptoms (Kish et al., 1988). Beside reduction of dopamine levels, PD also involves changes in neural volumes and connectivity among brain regions (Shine et al., 2013). This is accompanied with changes to medium spiny neurons in the striatum due to dopamine degeneration (Villalba and Smith, 2010). PD patients are often categorized into akinesia-dominant vs. tremor-domanient (Rajput et al., 2009; Zaidel et al., 2009; Moustafa et al., 2013a). It has been shown that motor symptoms in PD map to different neural dysfunctions. For example, akinesia and bradykinesia have been shown to be related to dopamine reduction in the basal ganglia (Albin et al., 1989; Spiegel et al., 2007; Rodriguez-Oroz et al., 2009; Rossi et al., 2010; Helmich et al., 2011), while tremor is related to abnormalities to the subthalamic nucleus, thalamus and cerebellum (Deuschl et al., 1998, 2000; Helmich et al., 2012).

To complicate the picture, tremor has been often divided into three subtypes: resting, action, and postural (Helmich et al., 2012). Another less common categorization method of tremor also includes a fourth category: intention tremor (Fahn, 2011). Other studies also categorize tremor based on its frequency (low or high) and body parts affected (hand, leg, or other). Some studies have suggested action and resting tremor can stem from dissociable neural systems (Raethjen et al., 2000; Cagnan et al., 2014). However, the exact mechanism of how neural abnormalities can generate either resting and action tremor is not known, but computational modeling work can shed light on these issues.

Other motor symptoms in PD were found to be impacted by other neural systems. For example, many patients with PD show freezing of gait, which is a feeling that the patient’s feet are glued to the ground. Studies have shown both dopamine and norepinephrine play a role in the occurrence of freezing of gait in PD patients (Bohnen et al., 2011; Lewitt, 2012). A recent computational model of freezing of gait in PD patients was able to shed a light on how alterations to these neurotransmitters impact gait function (Muralidharan et al., 2014). One limitation of this model, however, is it did not explain how connectivity loss among the cortex and basal ganglia can lead to freezing of gait.

Another common symptom in PD is dyskinesia, which is often associated with long-term use of dopamine medications (Bohnen et al., 2011), and occurs in over a quarter of patients with PD (Fabbrini et al., 2007). Many brain regions are implicated in the occurrence of dyskinesia, including the striatum or subthalamic nucleus (Soghomonian, 2006), prefrontal cortex (Cerasa et al., 2012), cerebellum (Kishore and Popa, 2014), and premotor cortex (Halje et al., 2012; Richter et al., 2013). Further, recent studies have suggested that dyskinesia in PD is likely related to abnormal changes in medium spiny neurons in the striatum (e.g., loss of dendritic spines), which is likely caused by impaired connectivity with dopamine neurons (Villalba and Smith, 2010; Suárez et al., 2014). This is in agreement with studies showing that both dopamine D1 and D2 receptors play a role in dyskinesia (Iravani et al., 2012). By using connectivity analyses, it was found that dyskinesia in PD is associated with altered connectivity between the striatum and primary motor cortex (Herz et al., 2015). Many neurotransmitters have been also implicated in the occurrence of dyskinesia in PD including serotonin, GABA, and glutamate (Bibbiani et al., 2005; Rylander et al., 2010; Politis et al., 2014). Interesting, it has been difficult to find treatment for dyskinesia in PD. However, it has been demonstrated that dopamine D1 receptors are crucially involved in levodopa-induced dyskinesia (Darmopil et al., 2009). In addition, a recent study has found that downstream regulatory element antagonistic modulator protein can also reduce levodopa-induced dyskinesia in Parkinsonian animals (Ruiz-DeDiego et al., 2015).

## Schizophrenia

Schizophrenia is a psychiatric disorder that can involve both positive symptoms (such as delusions and hallucinations) and negative symptoms (such as apathy, avolition and social withdrawal). Unlike other brain disorders, schizophrenia is often perceived to involve multiple and complex neural dysfunctions (Honea et al., 2005). These include dysfunction to the prefrontal cortex, basal ganglia, amygdala, and hippocampus (Cohen et al., 1996; Wright et al., 2000; Allen et al., 2011; Kühn et al., 2012; Yoon et al., 2013). It has been found that dysfunction of dopaminergic neurotransmission is widely observed in schizophrenia patients. However, dysregulation in other neurotransmission including serotonergic and GABAergic systems have been also reported (Carlsson et al., 2001; Abi-Dargham et al., 2002; Faghihi and Moustafa, 2015). Studies have shown reductions to regional brain volumes in patients with schizophrenia, including cortical gray matter (Cahn et al., 2002), medial temporal lobe and hippocampus (Wright et al., 2000; Honea et al., 2005). One study has found increased coupling between the posterior cingulate gyrus and precuneus in patients with schizophrenia (Pu et al., 2014). In contrast, few studies have dissociated positive and negative symptoms in schizophrenia (Buchanan et al., 1993; Abi-Dargham, 2003; Cascella et al., 2008; Arnedo et al., 2015). These studies suggested that positive symptoms may be related to dysfunction in the basal ganglia and hippocampus, while negative symptoms may be associated with neural damage to the prefrontal cortex (Wolkin et al., 1992).

It is important to note that hallucinations and delusions are often lumped together in one category, as measured by the positive and negative syndrome scale (PANSS; Moritz et al., 2001). However, based on studies from other patient populations, Poletti et al. (2012) found that delusions and hallucinations often do not co-exist in the same schizophrenia patient. To date, few studies have focused on subcomponents of positive symptoms in isolation. Bhatt et al. (2010) investigated cognitive dysfunction in schizophrenia patients with and without delusions; they found that the occurrence of delusions in schizophrenia is associated with more false memory errors, possibly suggesting that the hippocampus plays a role on both kinds of processes. Other studies have also investigated hallucinations in isolation (Hoffman et al., 2003; Schneider et al., 2011; Ford et al., 2014). For example, it has been found that hallucinations are associated with positive formal thought disorders (Sommer et al., 2010), suggesting that these two symptoms may share neurobiological substrates.

Using the psychiatric symptom rating scales (PSYRATS) allowing for dimensional assessment of hallucinations and delusions, Schneider et al. (2011), who dissociated the effects of psychiatric treatment on delusions vs. hallucinations in schizophrenia, found hallucinations tend to respond earlier to antipsychotic treatment than delusions. Other studies show that antipsychotic medications can reduce auditory hallucinations, while having minor effect on delusions (Lecrubier et al., 2007). Given the dissociable effects of treatments on hallucinations and delusions, it is likely that they are related to different neural abnormalities. It is possible that hallucinations and delusions are associated with separable neural systems, although some argue that delusions (false beliefs) may cause hallucinations (false perceptions; Maher, 1974; Ford et al., 2014). It is also possible that hallucinations are related to perceptual dysfunction (and thus impact posterior brain regions), while delusions are related to memory impairment (and thus related to temporal lobe dysfunction). Further, hallucinations are often broken down into subcategories (visual and auditory), as these present differently in various disorders, such as PD and schizophrenia. Further, it has also been argued that auditory hallucinations should be subgrouped into internally perceived (e.g., thought echo) vs. externally perceived (instructional voices; David, 2004), although the dissociable neural substrates of each is not known.

Further, although it was found depression and apathy are associated with different cognitive performances in PD (Pluck and Brown, 2002; Oguru et al., 2010; Kirsch-Darrow et al., 2011; Varanese et al., 2011), to our knowledge, dissociating the two symptoms was not conducted in schizophrenia studies. The findings that there are conflicting results of how different antipsychotics may impact depressive and negative symptoms in schizophrenia (Buckley and Stahl, 2007) could be due to differential effects of antipsychotics on different subcomponents of both general psychopathology and negative symptoms subscales (for discussion, see Lecrubier et al., 2007). In computational models of reinforcement learning, it has been shown that schizophrenia patients have impaired performance at acquisition of new knowledge on various reward and punishment learning tasks. This effect has been attributed to impairments of different aspects of the reward system (Somlai et al., 2011; Moustafa et al., 2015). More specifically, Corlett et al. (2010) have hypothesized that delusions arise from aberrations in how individuals compute prediction error—the mismatch between expectation and experience. They argue that defects in this fundamental brain mechanism in cortical and subcortical brain regions can vitiate perception, memory and social learning causing delusional thinking in psychotic illnesses.

## Posttraumatic Stress Disorder

PTSD is a fear and anxiety disorder, which develops in response to exposure to a traumatic event (e.g., physical and sexual assaults, combat experience, or motor vehicle accident) that produce fear responses triggered by subsequent exposures to reminders of the traumatic event (Gilbertson et al., 2008). Like other brain disorders, PTSD is characterized by the occurrence of different symptoms, including hyperarousal (e.g., problems concentrating, sleep problems), avoidance (e.g., avoiding any cues or contexts associated with the traumatic event), and re-experiencing (e.g., the recurrence of negative thoughts associated with the trauma; Gilbertson et al., 2008).

A wealth of data has shown that PTSD is associated with damage to the amygdala, ventromedial prefrontal cortex, hippocampus, and anterior cingulate (Gilbertson et al., 2008). Specifically, it has been found that a smaller hippocampal area is a risk factor for the development of PTSD (Gilbertson et al., 2002). Other studies have also reported increased activation levels in the amygdala, but reduced activation in the ventromedial prefrontal cortex in PTSD patients (Gilbertson et al., 2002). However, it remains unknown how these neural abnormalities relate to different symptoms of PTSD.

Few studies have attempted to dissociate the effects of PTSD symptoms, especially avoidance and re-experiencing. For instance, Myers et al. (2012) found that re-experiencing symptoms in PTSD is associated with impairment in generalization of learned rules using the acquired equivalence task (Kostek et al., 2014; Anastasides et al., 2015). This task has two phases: acquisition (learning to associate two stimuli) and transfer generalization (learning that cues become equivalent when they were previously associated with the same response). In this task, participants learn cues that were associated with the same feedback acquire a similarity such that subsequent generalization between these cues increases (Bondi et al., 1993). Myers et al. (2012) found that avoidance symptoms in PTSD are associated with behavioral inhibition. The same group applied a probabilistic classification task with trials resulting in reward, punishment and ambiguous outcomes (no-feedback response) to male veterans with severe and few or no PTSD symptoms. Those with severe PTSD symptoms outperformed the patients with few or no PTSD on reward-based trials, while there were no significant differences in punishment-based trials. Furthermore, patients with less severe PTSD symptoms rated ambiguous outcomes as more rewarding reflecting successful avoidance (Myers et al., 2013). Additionally, Hopper et al. (2007) also found that avoidance and re-experiencing symptoms are associated with different neural abnormalities, with avoidance severity negatively correlated with subcallosal anterior cingulate activity, while re-experiencing severity positively correlated with right anterior insula activity. Similarly, Contractor et al. (2013) revealed that some PTSD symptoms including dysphoria, avoidance and re-experiencing are related to alterations in the behavioral inhibition system that guides conflict resolution between approach and avoidance behaviors. To our knowledge, no study has focused on understanding the clinical and neural picture of hyperarousal in PTSD. Although there have been computational models of fear conditioning in PTSD (Moustafa et al., 2013b), none has attempted to understand the neural and behavioral correlates of different PTSD symptoms.

## Alzheimer’S Disease

AD is the most common form of dementia in old age, although it can also affect younger populations (Wilcock and Esiri, 1982). Dementia (including AD) is an umbrella term that involves loss of memory (semantic and episodic), apraxia, changes in language understanding and production, as well as executive dysfunction (Minati et al., 2009). Many of these symptoms are often subcategorized into additional subcomponents. For example, according to Cologne Apraxia Screening test, apraxia involves deficits to limb imitation, pantomime of object use, and imitation of face postures. One study suggested that apraxia has two components: commands to pantomime movements and mimicking movements (Foster et al., 1986).

Like most neurological and psychiatric disorders, AD is associated with multiple neural abnormalities mostly affecting the neocortex and hippocampal region (although there are reports of dysfunction to other brain regions). Additionally, AD is also associated with the formation of beta-amyloid plaques and neurofibrillary tangles in the cortex and hippocampal regions (Wilcock and Esiri, 1982) and a reduction of acetylcholine levels in the hippocampus and cortex (Wilcock and Esiri, 1982). Importantly, it is not known which neural damage gives rise to which symptoms in AD.

Anterograde amnesia in AD has been shown to be related to cortical abnormalities (Becker and Overman, 2002; Small et al., 2003). These findings are in agreement with data showing that the hippocampal region plays an important role in memory acquisition and consolidation, while the neocortex accounts for the maintenance of long-term memory (Squire and Alvarez, 1995). Several studies have investigated neural correlates of language processing in AD (Whitwell et al., 2015). One study, however, found that deficits in naming objects in AD are associated with reduced activation of inferior temporal lobes (Melrose et al., 2009).

To our knowledge, there is a dearth of studies investigating clinical and therapeutic correlates of different symptoms in AD, such as comparing memory severity to language dysfunction in AD. There is, however, one recent study that investigated neural correlates of subcomponents of apraxia, showing that unlike pantomiming of object-use, limb imitation processes are associated with underactivation to the inferior occipital gyrus (Johnen et al., 2015). Unlike PD, there are almost no studies that attempt to subcategorize AD patients into subgroups to investigate clinical, neural, and therapeutic aspects of each symptom in isolation. Further, unlike PD, there are fewer numbers of computational models of AD, and to our knowledge, few of them mainly focus on understanding memory decline and learning deficits in AD. For example, a network model by Ruppin and Reggia (1995) simulated impairment in retrieval of recent memories in AD. Tippett and Farah (1994) provided a feedforward connectionist model, and showed that naming objects in AD patients is related to semantic memory deficit in AD. Hasselmo (1997) provided a hippocampus model showing that deficits in memory encoding and retrieval in AD patients are possibly due to impaired pattern separation of new and old inputs in the hippocampus. In one study, deficits of acquired equivalent learning were tested in patients with mild AD (Bódi et al., 2009). Acquired equivalence, which is markedly impaired in case of hippocampal damage (Myers et al., 2003), refers to the phenomenon in which prior training to treat two stimuli as equivalent leads to generalization between them, even if the stimuli are characterized by superficial differences. Authors found that AD patients had mild impairments in the training phase; however, they presented profound deficits in the acquired equivalence task (Bódi et al., 2009).

## Conclusion

**Figure 1:**
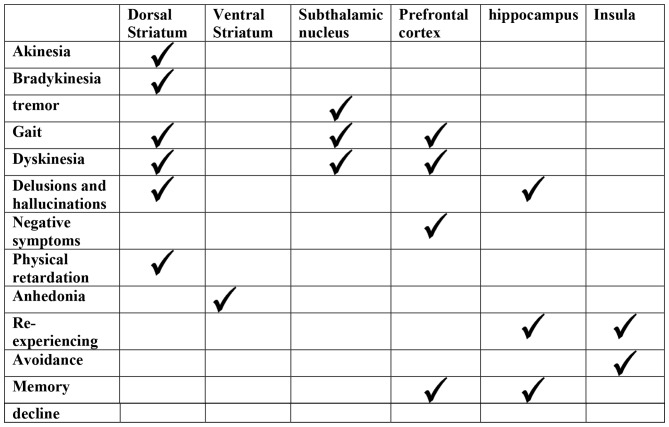
**A summary of the relationship between different symptoms in brain disorders discussed here, and corresponding neural abnormalities.** A tick mark means the corresponding brain area plays a role in this symptom, while empty cells means either it does not, or no existing data on their relationship.

This article recommends a symptom-based, rather than whole disease-based, approach to understand neural correlates of neurological and psychiatric disorders (see Figure 1 for a summary of results on the relationship between different symptoms and neural substrates). As discussed above, some studies have adopted this direction and investigated symptom clusters separately, such as tremor in PD (Hess and Pullman, 2012; Zhang et al., 2015), positive and negative symptoms in schizophrenia (Heckers et al., 1999), and psychomotor retardation in depression (Liberg and Rahm, 2015). Yet, this is not often the case for other symptoms or for other brain disorders, such as PTSD and AD. Importantly, the approach described here can be applied to other brain disorders, as most disorders involve damage to different neural systems and are associated with different clusters of symptoms. The challenge is to map neural damage to symptom clusters.

It is important to note that the different symptoms in a brain disorders are not necessarily dissociable (i.e., each is associated with different neural substrates and clinical profile). For example, some studies found that some motor symptoms in PD correlate with each other, including hand movement, gait and speech (Giladi et al., 2001; Goberman, 2005; Moreau et al., 2007; Cantiniaux et al., 2010; Naismith and Lewis, 2010; Nutt et al., 2011; Skodda et al., 2011; Vercruysse et al., 2012; Wagle Shukla et al., 2012). These correlations suggest that pharmacological drugs may affect each cluster of symptoms in a similar manner. Future studies should investigate the relationships among the different symptoms in a brain disorder; it is expected that related symptoms may share similar neural mechanism and may benefit from same treatment. For example, it was found that episodic memory and executive function processes correlate in AD (Baudic et al., 2006), but it is not known whether other symptoms in AD correlate with each other (including language production deficits and executive dysfunction). As we will discuss below, future computational models should attempt to provide a unified account of related symptoms, so they can provide a plausible neural mechanism for symptoms. Importantly, computational models can be used to provide best treatment for brain disorders (see for example, a model of treatment of schizophrenia symptoms by Siekmeier and vanMaanen, 2013).

A symptom-based approach will help us understand commonalities among disorders. For example, depression is co-morbid with many other disorders, including PD, schizophrenia, among others, and it is possible that these all share a common neural dysfunction. Depression in all these disorders have been associated with decreased similar neural mechanisms, including changes to dopamine and serotonin (Remy et al., 2005; Shen et al., 2012). This approach is also in line with the Research Domain Criteria (RDoC) project by the National Institute of Health (NIH), which proposes to study information processing mechanisms underlying single symptoms across different disorders. For example, akinesia in PD and psychomotor retardation in MDD are both associated with reduced dopamine levels in the striatum, thus suggesting both may share a similar mechanism.

Understanding the clinical picture and neural substrate of each symptom may also help the development of treatment for these symptoms. This may also help address conflicting results in the literature regarding the effects of pharmacological drugs on symptoms. For example, there have been conflicting results on whether antipsychotics can ameliorate negative symptoms in schizophrenia (Kane et al., 1988; Fitton and Heel, 1990; Miller et al., 1994; Schooler, 1994; Klemm et al., 1996; Risch, 1996; Rosenheck et al., 1997; Fink-Jensen, 2000; Rueter et al., 2004; Horacek et al., 2006; Tamrakar et al., 2006; Buckley and Stahl, 2007; Curtis et al., 2008). This could be due the fact that negative symptoms in schizophrenia are not monolithic constructs and may vary from individual from another. This same analysis can also be applied to other brain disorders.

It is important to note the process of subcategorizing a symptom into several components is an ongoing process, and future research is likely to further refine the subcategories of symptoms in brain disorders. For example, hallucinations and delusions are different classes of symptoms in schizophrenia, and hallucinations can be subcategorized into internally or externally perceived. The same applies to tremor, as it is often subcategorized based on type (into resting tremor, action, or postural tremor) or based on frequency (low or high) or body part affected (neck, hand, or leg). As another example, language deficits have been reported in AD, and it is likely that subcategorizing language deficits into several components may help understand the neural and therapeutic aspects of each in isolation. Similarly, as reported above, few studies have subcategorized apraxia in AD into motor processes that involve either mimicking or pantomiming, and report that these involve differential neural mechanisms (Foster et al., 1986).

Understanding the neural correlates of a symptom does not necessarily mean we understand its information processing mechanism. This is important as it may help developing a treatment for brain disorders (Siekmeier and vanMaanen, 2013). To do so, we must design a computational model to understand how damage to some neural systems lead to these symptoms. Computational models can aid in providing links of brain-behavioral relationships in relation to neuropsychological disorders. If dopamine is the main culprit in symptoms in PD, computational models should be able to provide a mechanistic account for how dopamine reduction cause these symptoms. Importantly, computational models should be able to explain how an increase or decrease in functional connectivity, regional brain volume, or neurotransmitter levels relates to symptoms in different disorders. For example, a model can explain how and why an increase (but not a decrease) in connectivity among brain regions or neurotransmitter leads to certain symptoms. Importantly, there have been some attempts to simulate different symptoms in brain disorders. For example, some computational models have simulated tremor in PD (Frank et al., 2007; Shaikh et al., 2010; Dovzhenok and Rubchinsky, 2012), gait dysfunction in PD (Muralidharan et al., 2014), hallucinations and psychosis in schizophrenia (Ermentrout and Cowan, 1979; Chen, 1995; Siekmeier et al., 2007; Lisman et al., 2010), avoidance (main symptom in PTSD; Sheynin et al., 2015), and language production deficits in AD (Conley et al., 2001). Future computational modeling work should focus on simulating other symptoms in these disorders as well as in other brain disorders.

There have been other ways of subcategorizing psychiatric and neurological disorders. For example, there are a few ways to categorize AD using genetics, as AD patients are often categorized in familial or sporadic, or into APOE ε4 carriers or not (Duara et al., 1993; van Duijn et al., 1995). Further, AD and PD are also often categorized into early vs. late-onset (younger or older than 65 in AD and younger or older than 50 in PD; Koedam et al., 2010) or based on age of onset (Foltynie et al., 2002). Other methods include disease duration and/or disease severity, which is usually done in AD and PD research as these are progressive disorders and symptoms change over time. Although these are very useful and relatively easy to do, subcategorizing a brain disorder based on symptoms has the additional following benefits: (a) a symptom is a behavioral trait and amenable to computational modeling and (b) many symptoms (e.g., depression and anxiety) co-occur in various brain disorders, so understanding these may help find treatment to them regardless of the disorder.

To sum up, for each brain disorder, future work in cognitive neuroscience, neuropsychology, and computational modeling should: (a) investigate clinical profile of the different symptoms; (b) study whether any of these correlate and thus related; (c) test the effects of drugs on each symptom in isolation; (d) investigate the neural substrates of each symptom; and (e) design computational models to understand the neural mechanism of each symptom in isolation. For example, a model of a brain region (e.g., hippocampus) should be able to explain all symptoms associated with hippocampus dysfunction, including memory decline in dementia and delusions in schizophrenia.

## Author Contributions

All authors listed, have made substantial, direct and intellectual contribution to the work, and approved it for publication.

## Conflict of Interest Statement

The authors declare that the research was conducted in the absence of any commercial or financial relationships that could be construed as a potential conflict of interest.
